# Effect of Deproteinization on the Clinical Success of Composite Restorations in Molars Affected by Molar–Incisor Hypomineralization

**DOI:** 10.3390/children13070858

**Published:** 2026-06-27

**Authors:** Necibe Damla Şahin, Beyza Günaydın

**Affiliations:** Department of Pediatric Dentistry, Faculty of Dentistry, Tokat Gaziosmanpaşa University, Tokat 60100, Türkiye; beyza.gunaydin@gop.edu.tr

**Keywords:** molar–incisor hypomineralization, sodium hypochlorite, deproteinization, composite restoration, survival analysis, USPHS criteria

## Abstract

**Highlights:**

**What is the main finding?**

- In this exploratory split-mouth study, NaOCl deproteinization was not associated with a statistically significant improvement in the clinical success or survival of composite restorations in MIH-affected molars during the 18-month follow-up period. High clinical success rates were observed in both groups according to modified USPHS criteria.

**What is the implications of the main finding?**

- Within the limitations of this study, routine NaOCl deproteinization before composite restoration in MIH-affected molars was not associated with additional short-term clinical benefits; however, clinically relevant differences cannot be definitively excluded because of the limited statistical power. Further long-term clinical studies with larger sample sizes are needed to clarify the role of deproteinization protocols in MIH management.

**Abstract:**

**Background/Objectives:** Molar–incisor hypomineralization (MIH) is associated with impaired enamel structure and increased risk of restoration failure. Sodium hypochlorite (NaOCl) deproteinization has been suggested to improve adhesive bonding to MIH-affected enamel; however, evidence regarding its clinical effectiveness remains limited. This study evaluated the effect of NaOCl deproteinization on the clinical success and survival of composite restorations in MIH-affected first permanent molars. **Methods:** This non-randomized split-mouth observational clinical study included 42 MIH-affected first permanent molars from 21 children aged 6–12 years. Teeth were allocated into two groups according to the restorative protocol applied: conventional adhesive restoration or NaOCl deproteinization before adhesive restoration. Restorations were evaluated at 6, 12, and 18 months using modified USPHS criteria. McNemar’s test was used for paired comparisons of categorical outcomes. Restoration survival was evaluated using a Cox proportional hazards regression model with patient-level robust cluster variance estimation to account for the split-mouth design. **Results:** Favorable clinical outcomes were observed in both groups at the 6-, 12-, and 18-month follow-up evaluations. No statistically significant differences were found between the NaOCl-treated and untreated groups for any modified USPHS parameter throughout the follow-up period (*p* > 0.05). Although higher failure rates for secondary caries and retention were observed in the NaOCl-treated group at 18 months, these differences were not statistically significant (*p* = 0.625 and *p* = 0.250, respectively). Survival analysis using a cluster-adjusted Cox regression model also demonstrated no statistically significant association between NaOCl application and restoration failure (HR = 1.22, 95% CI: 0.25–5.89; *p* = 0.808). **Conclusions:** In this exploratory study, NaOCl deproteinization did not demonstrate a statistically significant influence on the clinical success or survival of composite restorations in MIH-affected molars during the 18-month follow-up period. However, the limited statistical power of the study does not allow definitive exclusion of a clinically important difference between the treatment protocols. Therefore, these findings should be interpreted with caution. Additional long-term investigations with larger cohorts are warranted.

## 1. Introduction

Molar–incisor hypomineralization (MIH) is a developmental enamel abnormality affecting first permanent molars and frequently permanent incisors [1]. Clinically, affected teeth may present with demarcated opacities, hypersensitivity, post-eruptive enamel breakdown, and increased susceptibility to caries [2]. The global prevalence of MIH has been reported to be approximately 13.5%, although prevalence rates ranging from 2% to 40% have been reported among different populations [1]. Due to rapid enamel breakdown, increased treatment needs at an early age, hypersensitivity, and esthetic concerns, MIH is considered a significant public health problem that adversely affects oral health-related quality of life in children [2].

Enamel affected by MIH exhibits lower mineral content, altered organic matrix composition, and greater porosity compared with sound enamel. As a result, adhesive systems may have difficulty achieving effective micromechanical retention on these surfaces [3]. Additionally, previous studies have reported that the typical etching pattern observed after acid conditioning appears less distinct and more irregular in MIH-affected enamel, while the porous enamel structure may further compromise bonding performance. Clinically, these characteristics have been associated with increased failure rates and retreatment needs of resin-based restorations in teeth affected by MIH [4].

To improve resin adhesion in teeth with enamel defects, deproteinization protocols aimed at removing the organic matrix from the enamel surface have been proposed [3,5]. Previous evidence syntheses have suggested that although adhesive bonding to hypomineralized enamel is generally weaker than that to sound enamel, deproteinization performed prior to acid etching may enhance bonding performance [6,7]. 

Sodium hypochlorite (NaOCl) is the most extensively investigated deproteinizing agent for developmental enamel defects and is commonly applied at concentrations of 5–5.25% for short periods to remove organic tissue from the enamel surface [3,8]. Systematic reviews focusing on MIH-affected enamel have reported that deproteinization with 5% NaOCl may improve bonding performance in many cases; however, the available findings remain inconsistent, and further clinical evidence is still required [3,8,9]. While several laboratory and clinical studies have reported favorable effects of NaOCl deproteinization on bond strength and restoration performance [3,5,10], other investigations have demonstrated limited or non-significant improvements, suggesting that the effectiveness of NaOCl may vary depending on lesion severity, treatment protocol, and concomitant restorative procedures [4,11]. In recent years, alternative deproteinizing agents such as papain-based formulations, hypochlorous acid (HOCl), and various oxidizing agents have also been investigated, with promising results reported particularly for dentin and smear layer modification. Nevertheless, sodium hypochlorite remains the most commonly used deproteinizing solution for MIH-affected enamel and continues to be the agent with the greatest amount of favorable evidence reported in the literature [12,13].

To date, a standardized bonding protocol capable of improving the long-term clinical success of composite restorations in children with MIH has not yet been established [3,5]. Although several in vitro studies have investigated the relationship between NaOCl deproteinization and bonding to hypomineralized enamel, clinical evidence remains limited. Furthermore, most of the currently available evidence is derived from in vitro studies, and the heterogeneity in study protocols and outcome measures makes direct clinical interpretation challenging. In particular, only a small number of studies have evaluated the clinical performance of composite restorations in children affected by MIH [3,14,15]. 

To the best of our knowledge, clinical studies investigating the effect of NaOCl deproteinization on the success of composite restorations in MIH-affected teeth using a split-mouth design are still scarce. Therefore, the present study aimed to evaluate the effect of sodium hypochlorite deproteinization on the clinical success of composite restorations in children with molar–incisor hypomineralization using a split-mouth design. It was considered that the findings of this study may contribute to the development of evidence-based clinical protocols for the restorative management of MIH-affected teeth. The null hypothesis of the study was that there would be no difference in the 18-month clinical success of composite restorations performed with and without NaOCl deproteinization in MIH-affected teeth.

## 2. Materials and Methods

**Study Design and Ethical Approval:** This study was designed as a split-mouth observational clinical study conducted at the Department of Pediatric Dentistry, Faculty of Dentistry, Tokat Gaziosmanpaşa University. Baseline data were obtained retrospectively, whereas the clinical follow-up evaluations of the restorations were performed prospectively. Ethical approval was obtained from the Non-Interventional Clinical Research Ethics Committee of Tokat Gaziosmanpaşa University Faculty of Medicine (Approval No: 25-MOBAEK-284, approval date: 2 September 2025). Written informed consent was obtained from the parents or legal guardians of all participating children, and verbal assent appropriate to the children’s age was also obtained. The study was conducted according to the Declaration of Helsinki principles.

**Patient Selection and Sample Size:** The study population consisted of children aged 6–12 years who were diagnosed with molar–incisor hypomineralization (MIH) and attended the clinic between February 2024 and May 2024. Initial patient data were retrospectively obtained from the digital patient record system used at the faculty. The study sample was composed of all eligible patients who met the inclusion criteria within the specified study period. Due to the retrospective baseline design of the study, a priori sample size calculation was not performed. Therefore, a post hoc power assessment was conducted using the observed event rates for the primary outcome (retention) at the 18-month follow-up. At an alpha level of 0.05, the achieved statistical power was calculated as 28.4%, indicating that the study was underpowered to detect small-to-moderate differences between groups. All restorative procedures included in the study were carried out by the same clinician.

**Inclusion Criteria:**

- Children aged 6–12 years;
- Absence of systemic disease;
- Frankl Behavior Rating Scale score of 3 or 4;
- Bilateral MIH involvement affecting the permanent first molars in either the maxilla or mandible;
- MIH-affected first permanent molars classified as MIH-TNI 2b or 4b [16], involving more than one-third but less than two-thirds of the tooth surface and presenting deep dentin involvement requiring restorative treatment without spontaneous pain;
- Deep dentin caries suitable for composite restoration, with no history of spontaneous or acute pain;
- Teeth considered suitable for conservative restorative treatment; when clinically indicated, conservative vital pulp therapy procedures (indirect pulp capping or partial pulpotomy) were performed prior to restoration;
- Availability for regular follow-up evaluations at 6, 12, and 18 months;
- Complete clinical evaluation records based on the modified USPHS criteria;
- All restorative procedures performed by the same clinician.

**Exclusion Criteria:**

- Teeth requiring extensive pulp therapy or endodontic treatment;
- Teeth not meeting the specified inclusion criteria.

During the data evaluation process, two experienced pediatric dentists independently assessed 10 randomly selected patient records in two separate sessions conducted at a two-week interval to standardize the inclusion criteria assessment. Inter-examiner agreement was found to be excellent (Cohen’s kappa = 0.90).

**Treatment Procedures and Study Groups:** According to the split-mouth design, MIH-affected teeth belonging to the same patient were allocated into two different groups. During the treatment process, the decision regarding which tooth would receive deproteinization was made by the operator at the time of the clinical procedure. All clinical procedures were performed by the same investigator.

Following local anesthesia administration, cavity preparation was performed using a high-speed handpiece under water cooling, while carious dentin tissue was removed with low-speed burs. Enamel areas affected by MIH were included within the cavity margins, and unsupported hypomineralized enamel was removed. Throughout all procedures, the operative field was isolated using a rubber dam to ensure proper isolation.

The study groups were defined as follows:

- **Group 1 (Control):** Acid etching + adhesive system + composite restoration;
- **Group 2 (Experimental):** 5% NaOCl deproteinization + acid etching + adhesive system + composite restoration.

In Group 2, deproteinization was performed by applying a 5% sodium hypochlorite (NaOCl) solution to the enamel surface following cavity preparation and isolation. The NaOCl solution was applied for 1 min, after which the surface was rinsed with water spray and air-dried. Subsequently, the enamel surface was acid-etched with 37% phosphoric acid (RubyEtch, RubyDent, İstanbul, Turkey) for 30 s.

Subsequently, a universal adhesive system (Prime&Bond Universal, Dentsply Sirona, Konstanz, Germany) was applied in all groups according to the manufacturer’s instructions and light-cured using an LED curing unit (Woodpecker LED B, Guilin Woodpecker Medical Instrument Co., Guilin, China). Composite resin material (NeoSpectra™, Dentsply Sirona, Charlotte, NC, USA) was then placed incrementally into the cavity, and each increment was polymerized for 20 s. Finishing and polishing procedures were performed using a finishing and polishing disc system (Enhance^®^ Finishing System and Enhance^®^ PoGo^®^ Polishing System, Dentsply Sirona, Charlotte, NC, USA). Regarding the clinical procedures performed, 17 teeth received composite restoration alone, 15 teeth underwent indirect pulp capping, and 10 teeth received partial pulpotomy prior to restoration. No direct pulp capping or full pulpotomy procedures were performed in the present study.

**Clinical Evaluation:** The restorations were clinically evaluated at the 6-, 12-, and 18-month follow-up periods using modified USPHS criteria, including retention, marginal adaptation, marginal discoloration, secondary caries, surface roughness, color match, and postoperative sensitivity (Table 1). As all clinical procedures were performed by a single investigator, operator blinding was not feasible. However, the clinical evaluation of the restorations was carried out by an independent examiner who was blinded to the treatment protocol and unaware of the group allocation. This approach was adopted to minimize observer bias during the evaluation process. To ensure the reliability of the assessments, intra-examiner agreement was evaluated during the data analysis phase. For this purpose, a selected number of restorations were re-evaluated after a two-week interval, and a high level of agreement was observed (Cohen’s kappa = 0.90). For all evaluation criteria, Alpha and Bravo scores were considered successful, whereas Charlie scores were regarded as failures. Each USPHS criterion was evaluated independently, and an unsuccessful score in one parameter did not automatically result in unsuccessful scores in other parameters (see Supplementary Table S1).

**Statistical Analysis:** Statistical analyses were performed using IBM SPSS Statistics for Windows, Version 27.0 (IBM Corp., Armonk, NY, USA). Continuous variables were expressed as mean ± standard deviation (SD), mean ± standard error (SE) with 95% confidence intervals (CI), or median (minimum–maximum) where appropriate, while categorical variables were presented as frequencies (n) and percentages (%). Because the study employed a split-mouth design in which multiple restorations from the same patient were evaluated, paired and cluster-adjusted, analyses were used to account for within-patient correlation. Clinical outcomes according to the modified USPHS criteria were compared between NaOCl-treated and untreated restorations using McNemar’s test. For survival analyses, Kaplan–Meier methods were used to estimate restoration survival over time. To account for the clustering of restorations within the same patient, the association between NaOCl application and restoration failure was evaluated using a Cox proportional hazards regression model with patient-level robust cluster variance estimation (sandwich estimator). For survival analysis, restoration failure was defined as the first occurrence of a Charlie score in any of the evaluated modified USPHS criteria. The time of failure was recorded as the follow-up visit (6, 12, or 18 months) at which the first Charlie score was detected. Restorations that did not receive a Charlie score during the observation period were considered censored at their last available follow-up assessment. Survival times were reported as mean ± SE with 95% CI, and hazard ratios (HRs) were presented with their corresponding 95% CIs. A two-tailed *p* value of <0.05 was considered statistically significant for all analyses.

## 3. Results

**Figure 1:**
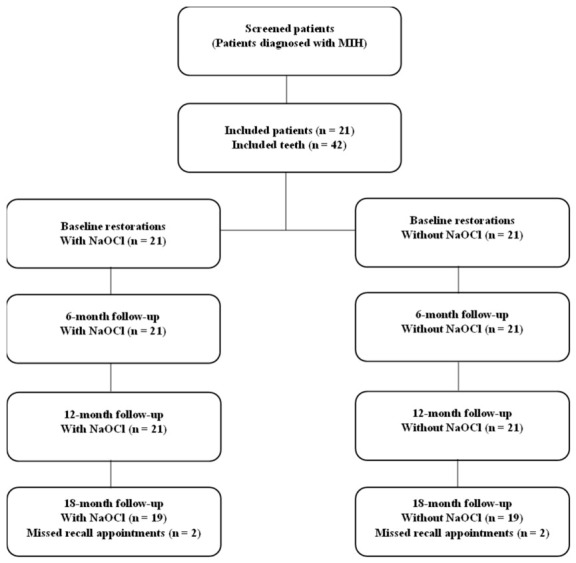
Flow diagram of the included patients and restorations during the 18-month follow-up period.

A total of 42 MIH-affected teeth from 21 patients were included in the study. Because the study employed a split-mouth design, paired and cluster-adjusted statistical methods were used to account for within-patient correlation. The follow-up flow of the included patients and restorations is presented in Figure 1. During the follow-up period, two restorations in the NaOCl group and two restorations in the control group were lost to follow-up before the 18-month evaluation because the patients did not attend the scheduled recall visits. These restorations were treated as censored observations in the survival analysis. Of the evaluated teeth, 71.4% belonged to female patients and 28.6% to male patients. The median age of the participants was 8.67 years (minimum–maximum: 6.17–13.42 years). Among the included teeth, 61.9% were maxillary first permanent molars (16–26), whereas 38.1% were mandibular first permanent molars (36–46). Demographic characteristics and tooth distribution are presented in Table 2.

Baseline distributions of initial sensitivity and pulpal procedures according to NaOCl application are presented in Table 3. No statistically significant differences were observed in the available baseline variables. However, because lesion-level characteristics such as opacity type, post-eruptive enamel breakdown, and lesion severity were not systematically recorded, residual baseline differences cannot be excluded. McNemar analyses included 21 evaluable pairs at the 6- and 12-month follow-up evaluations and 19 evaluable pairs at the 18-month follow-up evaluation because two patients were lost to follow-up.

High success rates were observed for all evaluated parameters according to the modified USPHS criteria at the 6-, 12-, and 18-month follow-up evaluations. No statistically significant differences were found between the NaOCl-treated and untreated groups regarding color match, marginal adaptation, anatomical form, surface roughness, marginal discoloration, postoperative sensitivity, secondary caries, or retention at any evaluation period (*p* > 0.05 for all comparisons).

At the 18-month evaluation, higher failure rates for secondary caries and retention were observed in the NaOCl-treated group; however, these differences were not statistically significant (*p* = 0.625 and *p* = 0.250, respectively). The clinical success results at the 6-, 12-, and 18-month follow-up periods according to NaOCl application are presented in Table 4.

**Figure 2:**
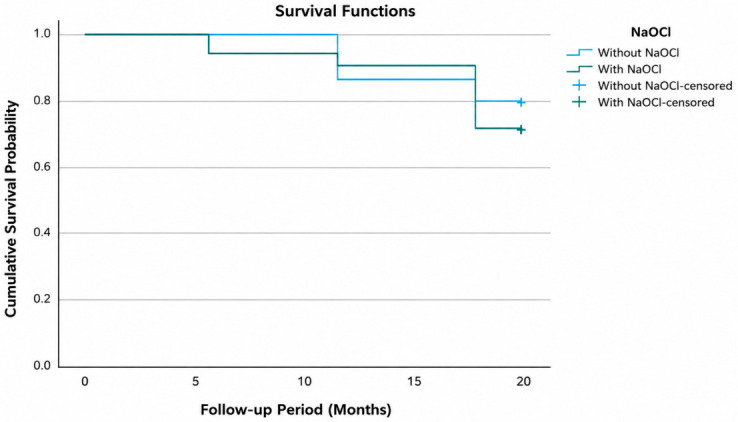
Kaplan–Meier survival curves of restorations in the NaOCl-treated and untreated groups during the 18-month follow-up period.

No statistically significant association was observed between NaOCl application and restoration failure risk during the 18-month follow-up period. In a Cox proportional hazards regression model with patient-level robust cluster variance estimation to account for within-patient correlation, NaOCl application was not significantly associated with restoration survival (HR = 1.22, 95% CI: 0.25–5.89, *p* = 0.808). However, the wide confidence interval indicates substantial uncertainty regarding the magnitude and direction of the estimated treatment effect. Mean survival times were similar between the groups (Table 5). Kaplan–Meier survival curves are presented in Figure 2 and demonstrated comparable survival patterns in the NaOCl-treated and untreated groups throughout the follow-up period.

## 4. Discussion

Restorative management of MIH-affected molars remains clinically challenging due to the hypomineralized enamel structure, and previous studies have reported higher restoration failure rates compared with sound enamel [3,4,17]. Therefore, deproteinization procedures aimed at removing the organic matrix from the enamel surface have been proposed to improve adhesive bonding performance. Nevertheless, there is still no clear consensus in the literature regarding the clinical effectiveness of this approach [6,13,18,19]. In the present study, the effect of NaOCl deproteinization on the clinical success and survival of composite restorations in MIH-affected first permanent molars was evaluated over an 18-month follow-up period using a split-mouth design and modified USPHS criteria. The findings of this non-randomized split-mouth observational study did not demonstrate a statistically significant association between NaOCl application and the clinical performance of the restorations. Accordingly, the null hypothesis could not be rejected.

The split-mouth design used in the present study allowed different treatment modalities to be compared within the same individual while minimizing the influence of patient-related variables such as oral hygiene, salivary composition, and dietary habits [20]. Nevertheless, because treatment allocation was determined clinically by the operator and lesion-level baseline characteristics, particularly lesion severity, were not systematically recorded, residual confounding and selection bias related to lesion severity and operator allocation cannot be completely excluded. Although no statistically significant differences were observed in the available baseline variables, non-significant *p*-values do not confirm that the groups were adequately balanced. Residual differences in unmeasured variables, particularly lesion severity, may persist and could influence both treatment allocation and outcomes. Restoration performance was assessed using modified USPHS criteria, a widely accepted system enabling standardized evaluation of parameters such as retention, marginal adaptation, surface roughness, and secondary caries, while facilitating comparison with previous clinical studies [19,21].

High success rates according to the modified USPHS criteria were observed in both groups at the 6-, 12-, and 18-month follow-up evaluations, suggesting that composite restorations performed using appropriate adhesive protocols may provide acceptable short- to medium-term clinical performance in MIH-affected molars [17,19,22]. In the present study, no statistically significant differences were found between NaOCl-treated and untreated teeth at any evaluation period (*p* > 0.05). Similarly, Ozsoy and Erken Gungor [21] reported no significant difference between deproteinized and non-deproteinized groups in their clinical study. Moreover, several in vitro studies have also demonstrated that NaOCl application may not significantly improve bond strength. Previous studies investigating the effect of NaOCl deproteinization in MIH-affected enamel have reported inconsistent findings. While Chay et al. [15], Ekambaram et al. [8], and clinically Sönmez et al. [14] reported improved bond strength or restoration survival following NaOCl application, Gandhi et al. [23] and Krämer et al. [4] observed limited or non-significant improvements in enamel bonding performance. It has further been suggested that the sequence of application, particularly whether deproteinization is performed before or after acid etching, may play a critical role in the resulting bonding performance [4,23]. Therefore, the findings of the present study suggest that, within the current sample size and follow-up period, NaOCl deproteinization did not demonstrate a statistically significant advantage. Nevertheless, numerical differences observed at the 18-month follow-up, particularly regarding retention and secondary caries, should be interpreted cautiously. Although these findings did not reach statistical significance, potential clinical implications cannot be completely excluded and may become more apparent in studies with larger sample sizes and longer follow-up periods.

Nevertheless, some in vitro studies have reported that NaOCl application may enhance the bond strength of adhesive systems to MIH-affected enamel. It has also been suggested that this effect may depend on the lesion type, being more pronounced in cream-white opacities while remaining limited in yellow-brown lesions [8,15]. However, the in vitro nature of these studies should be taken into consideration, as they cannot fully reproduce the dynamic conditions of the oral environment, including saliva, thermal fluctuations, and mechanical loading. In a previous clinical study, NaOCl application was reported to improve restoration survival after a 24-month follow-up period, although no significant differences between the groups were observed during the first 12 months [14]. This finding may indicate that the potential effects of NaOCl are more likely to become evident over longer follow-up periods rather than during short-term clinical evaluation.

The variable findings reported in the literature may partly be explained by the heterogeneous nature of MIH lesions. Differences in porosity, protein content, mineralization degree, adhesive systems, and clinical protocols may directly influence the response to deproteinization procedures and restorative outcomes.

The similar survival times observed in both groups suggest that NaOCl application did not demonstrate a statistically significant influence on restoration survival during the 18-month follow-up period. However, the wide confidence interval associated with the hazard ratio estimate indicates substantial uncertainty regarding the magnitude and direction of the potential treatment effect. This finding is consistent with previous studies suggesting that restoration survival in MIH-affected molars may be influenced more by material type, cavity design, and lesion severity than by a single surface pretreatment protocol [17,22,24].

This study has several limitations that should be considered. First, the relatively small sample size may have limited the statistical power of the analyses. Consistent with this, the post hoc power analysis demonstrated limited statistical power, suggesting that small-to-moderate differences between treatment groups may not have been detected. Therefore, the findings should be interpreted with caution, and the limited statistical power does not allow definitive exclusion of a clinically important difference between the treatment groups. Additionally, the 18-month follow-up period does not allow assessment of long-term clinical performance. Although an attempt was made to reduce lesion heterogeneity by including MIH-affected molars with comparable lesion extent according to the MIH-TNI classification, formal lesion severity stratification was not performed. Furthermore, lesion distribution according to MIH-TNI subcategories within each treatment group was not systematically recorded during the retrospective baseline phase of the study and therefore could not be evaluated retrospectively. Furthermore, lesion distribution according to MIH-TNI subcategories within each treatment group was not systematically recorded during the retrospective baseline phase of the study and therefore could not be evaluated retrospectively. Because lesion severity was not systematically recorded and treatment allocation was operator-determined, residual confounding by lesion severity and selection bias cannot be ruled out. This is a fundamental limitation of the non-randomized design. Moreover, operator blinding was not feasible due to the nature of the intervention, and therefore a potential risk of operator-related bias cannot be completely excluded. In addition, detailed lesion presentation characteristics, such as opacity type and post-eruptive enamel breakdown, were not systematically recorded, although these factors may directly influence restoration performance in MIH-affected teeth. Finally, secondary caries assessment was based solely on clinical examination. Because interproximal radiographs were not routinely available in this retrospective study, early proximal secondary caries lesions may have been underestimated. Furthermore, the number of restored surfaces and detailed cavity extent were not systematically recorded due to the retrospective baseline nature of the study, which may limit reproducibility and detailed interpretation of the restorative procedures. Although the split-mouth design reduced patient-related variability, potential intra-patient correlation between teeth cannot be completely excluded. Consequently, a degree of non-independence between observations may have influenced the statistical findings and should be considered when interpreting the results. Nevertheless, the use of a split-mouth design represents one of the strengths of the present study, as it enabled better control of individual-related variables.

## 5. Conclusions

Within the limitations of this exploratory, underpowered, non-randomized split-mouth observational study, NaOCl deproteinization was not associated with a statistically significant difference in the clinical success of composite restorations in MIH-affected teeth during the 18-month follow-up period. However, a clinically important benefit or harm cannot be excluded because of the limited statistical power and the potential influence of residual confounding. Although numerical differences were observed in certain clinical outcomes, particularly retention and secondary caries at the 18-month evaluation, these findings should be interpreted cautiously. Further well-designed clinical studies with larger sample sizes and longer follow-up periods are warranted.

## Figures and Tables

**Table 1:** Modified USPHS Criteria Used in the Study.

Parameter	Score	Clinical Condition
Color match	Alpha	Restoration compatible with surrounding tooth structure
	Bravo	Slight color mismatch, clinically acceptable
	Charlie	Obvious color mismatch, clinically unacceptable
Marginal adaptation	Alpha	Margins closely adapted, no visible crevice
	Bravo	Visible crevice detectable with an explorer
	Charlie	Crevice with exposed dentin
Anatomical form	Alpha	Restoration continuous with the original tooth anatomy
	Bravo	Slight discontinuity, clinically acceptable
	Charlie	Discontinuous restoration, clinically unacceptable
Surface roughness	Alpha	Enamel-like surface
	Bravo	Slightly rough surface, clinically acceptable
	Charlie	Unacceptably rough surface
Marginal discoloration	Alpha	No discoloration
	Bravo	Superficial discoloration
	Charlie	Deep discoloration
Postoperative sensitivity	Alpha	No sensitivity
	Bravo	Mild sensitivity
	Charlie	Severe sensitivity
Secondary caries	Alpha	No caries present
	Charlie	Caries present
Retention	Alpha	No loss of restoration
	Charlie	Loss of restoration

**Table 2:** Demographic Characteristics and Tooth Distribution.

		n (%)
Sex	Female	15 (71.4)
	Male	6 (28.6)
Tooth distribution	16–26	26 (61.9)
	36–46	16 (38.1)
Age, Median (min–max)		8.67 (6.17–13.42)

**Table 3:** Baseline clinical characteristics according to treatment group.

Variable	Without NaOCl n (%)	With NaOCl n (%)	*p* Value
Initial sensitivity			0.719
Present	4 (19.0)	6 (28.6)	
Absent	17 (81.0)	15 (71.4)	
Pulpal procedure			0.720
Composite restoration only	12 (57.1)	9 (42.9)	
Indirect pulp capping	5 (23.8)	6 (28.6)	
Partial pulpotomy	4 (19.0)	6 (28.6)	

Data are presented as n (%). Fisher’s exact test was used for initial sensitivity, and Fisher–Freeman–Halton Exact test was used for pulpal procedure distribution. NaOCl, sodium hypochlorite.

**Table 4:** Clinical Success Outcomes at 6-, 12-, and 18-Month Follow-up Evaluations According to NaOCl Application.

Parameter	Outcome	6 Months Without NaOCl n (%)	6 Months With NaOCl n (%)	12 Months Without NaOCl n (%)	12 Months With NaOCl n (%)	18 Months Without NaOCl n (%)	18 Months With NaOCl n (%)
Color match	Successful	21 (100)	20 (95.2)	20 (95.2)	20 (95.2)	18 (94.7)	18 (94.7)
	Unsuccessful	0 (0)	1 (4.8)	1 (4.8)	1 (4.8)	1 (5.3)	1 (5.3)
	*p* value	1.000		1.000		1.000	
Marginal adaptation	Successful	21 (100)	20 (95.2)	21 (100)	20 (95.2)	19 (100)	18 (94.7)
	Unsuccessful	0 (0)	1 (4.8)	0 (0)	1 (4.8)	0 (0)	1 (5.3)
	*p* value	1.000		1.000		1.000	
Anatomical form	Successful	21 (100)	20 (95.2)	21 (100)	20 (95.2)	19 (100)	18 (94.7)
	Unsuccessful	0 (0)	1 (4.8)	0 (0)	1 (4.8)	0 (0)	1 (5.3)
	*p* value	1.000		1.000		1.000	
Surface roughness	Successful	21 (100)	20 (95.2)	21 (100)	20 (95.2)	19 (100)	18 (94.7)
	Unsuccessful	0 (0)	1 (4.8)	0 (0)	1 (4.8)	0 (0)	1 (5.3)
	*p* value	1.000		1.000		1.000	
Marginal discoloration	Successful	21 (100)	20 (95.2)	21 (100)	20 (95.2)	19 (100)	18 (94.7)
	Unsuccessful	0 (0)	1 (4.8)	0 (0)	1 (4.8)	0 (0)	1 (5.3)
	*p* value	1.000		1.000		1.000	
Postoperative sensitivity	Successful	21 (100)	20 (95.2)	21 (100)	20 (95.2)	19 (100)	18 (94.7)
	Unsuccessful	0 (0)	1 (4.8)	0 (0)	1 (4.8)	0 (0)	1 (5.3)
	*p* value	1.000		1.000		1.000	
Secondary caries	Successful	21 (100)	20 (95.2)	21 (100)	20 (95.2)	18 (94.7)	16 (84.2)
	Unsuccessful	0 (0)	1 (4.8)	0 (0)	1 (4.8)	1 (5.3)	3 (15.8)
	*p* value	1.000		1.000		0.625	
Retention	Successful	21 (100)	20 (95.2)	21 (100)	20 (95.2)	18 (94.7)	15 (78.9)
	Unsuccessful	0 (0)	1 (4.8)	0 (0)	1 (4.8)	1 (5.3)	4 (21.1)
	*p* value	1.000		1.000		0.250	

McNemar’s test was used for paired comparisons between groups. *p*-values are reported only when the test was mathematically estimable. Analyses at the 18-month evaluation were performed on 38 restorations due to the loss of four restorations to follow-up.

**Table 5:** Survival Analysis Results of Restorations According to NaOCl Application.

Group	n	Failure n (%)	Mean Survival Time (months) ± SE (95% CI)	HR	95% CI	*p* Value
Without NaOCl	21	4 (19.0)	17.43 ± 0.38 (16.68–18.18)	Reference	–	–
With NaOCl	21	5 (23.8)	17.43 ± 0.56 (16.34–18.52)	1.22	0.25–5.89	0.808

SE, standard error; CI, confidence interval; HR, hazard ratio. HR and 95% CI were derived from a Cox proportional hazards regression model using patient-level robust cluster variance estimation (sandwich estimator) to account for within-patient correlation.

## Data Availability

The data presented in this study are available from the corresponding author upon reasonable request.

## Supplementary Materials

The following supporting information can be downloaded at: https://www.mdpi.com/article/10.3390/children13070858/s1, Table S1: Distribution of Charlie scores according to USPHS criterion, treatment group, and follow-up period.
