# Supplementary material for: Effect of Deproteinization on the Clinical Success of Composite Restorations in Molars Affected by Molar–Incisor Hypomineralization
Source: Children (Basel). 2026 Jun 27;13(7):858. doi: 10.3390/children13070858 (PMC13406727; doi:10.3390/children13070858)
Supplement: Supplementary file 1 [file children-13-00858-s001.zip › children-4341742-supplementary.pdf]

Supplementary Table S1. Distribution of Charlie Scores According to USPHS Criterion, Treatment Group, and Follow-up Period.

Criterion	6 Months Without NaOCl	6 Months With NaOCl	12 Months Without NaOCl	12 Months With NaOCl	18 Months Without NaOCl	18 Months With NaOCl
**Color match**	0	1	1	1	1	1
**Marginal adaptation**	0	1	0	1	0	1
**Anatomical form**	0	1	0	1	0	1
**Surface roughness**	0	1	0	1	0	1
**Marginal discoloration**	0	1	0	1	0	1
**Postoperative sensitivity**	0	1	0	1	0	1
**Secondary caries**	0	1	0	1	1	3
**Retention**	0	1	1	1	1	4

Charlie scores are presented by criterion and follow-up period. The same restoration could contribute to more than one Charlie score across different criteria and/or follow-up evaluations. Restoration failure in the survival analysis was defined by the first occurrence of any Charlie score.
